# Blood pressure after PREeclampsia/HELLP by SELF monitoring (BP-PRESELF): rationale and design of a multicenter randomized controlled trial

**DOI:** 10.1186/s12905-020-00910-0

**Published:** 2020-03-04

**Authors:** Hella E. C. Muijsers, Olivier W. H. van der Heijden, Karin de Boer, Chantal van Bijsterveldt, Ciska Buijs, Jens Pagels, Peter Tönnies, Susanne Heiden, Nel Roeleveld, Angela H. E. M. Maas

**Affiliations:** 1 Department of Cardiology, Radboud university medical center, Geert-Grooteplein Zuid 10, 6525 GA Nijmegen, The Netherlands; 2 Department of Obstetrics and Gynecology, Radboud university medical center, Geert-Grooteplein Zuid 10, 6525 GA Nijmegen, The Netherlands; 3 Department of Obstetrics and Gynecology, Rijnstate, Wagnerlaan 55, 6815 AD Arnhem, The Netherlands; 4 Department of Obstetrics and Gynecology, Canisius-Wilhelmina Hospital, Weg door Jonkerbos 100, 6532 SZ Nijmegen, The Netherlands; 5 Department of Obstetrics and Gynecology, Maasziekenhuis Pantein, Dokter Kopstraat 1, 5835 BV, Beugen, The Netherlands; 6 Department of Obstetrics and Gynecology, St. Josef Hospital Moers, Asberger Strasse 4, 47441 Moers, Germany; 7 Department of Obstetrics and Gynecology, Bethanien Hospital Moers, Bethanienstrasse 21, 47441 Moers, Germany; 8 Department of Obstetrics and Gynecology, St. Antonius Hospital Kleve, Albersallee 5-7, 47533 Kleve, Germany; 9 Department for Health Evidence, Radboud Institute for Health Sciences, Radboud university medical center, Geert-Grooteplein Zuid 10, 6525 GA Nijmegen, The Netherlands

**Keywords:** Cardiovascular disease, eHealth, Preeclampsia, Hypertension, Home blood pressure monitoring, Cardiovascular risk factor

## Abstract

**Background:**

Hypertensive disorders of pregnancy (HDP), such as preeclampsia (PE) or the Hemolysis Elevated Liver enzymes and Low Platelets (HELLP) syndrome are associated with elevated cardiovascular disease (CVD) risks, but standardized prevention guidelines after such pregnancies are lacking. Hypertension is the first emerging risk factor after PE/HELLP pregnancies and is a major risk factor for CVD. Hypertension before the age of 55 years may lead to various manifestations of end-organ damage at relatively young age. Therefore, timely treatment of elevated blood pressure is mandatory, but many of these high-risk women have long-term undetected and untreated hypertension before adequate treatment is initiated.

**Aim:**

The aim of our study is to assess whether home blood pressure monitoring (HBPM) in women with a previous PE/HELLP pregnancy is a valuable tool for the early detection of hypertension.

**Methods:**

Women with a history of both early and late PE/HELLP syndrome aged 40–60 years are invited to participate. Patients with a history of CVD, known hypertension and/or use of antihypertensive medication are excluded. Women are randomized between HPBM or ‘usual care’. The primary outcome is feasibility and usability of HBPM after 1 year of follow-up.

Secondary outcomes will be the effectiveness of HPBM to detect hypertension, the efficacy of BP treatment, quality of life, health-related symptoms, work ability, and life-style behaviour.

The results of this study will provide better strategies for timely detection and prevention of hypertension in women after PE/HELLP.

**Trial registration:**

ClinicalTrials.gov, NCT03228082. Registered June 15, 2017.

## Background

Cardiovascular disease (CVD) is the leading cause of mortality in women worldwide. One of the major risk factors is high blood pressure [1]. In women, 38% of cardiovascular mortality could be prevented if this risk factor could be eliminated [2]. Important female-specific risk factors for CVD are hypertensive disorders of pregnancy (HDP), which may induce earlier occurrence of high blood pressure [3].

HDP complicate 10–15% of all pregnancies [4, 5]. This group consists of chronic hypertension, gestational or pregnancy-induced hypertension (PIH), preeclampsia (PE) and Hemolysis Elevated Liver enzymes Low Platelets (HELLP) syndrome. De novo hypertension during pregnancy is diagnosed when hypertension is present after 20 weeks of gestation.

PE and HELLP syndrome represent the most severe HDP with the highest CVD risk and complicate 3–5% of all pregnancies [6–8]. PE is diagnosed when gestational hypertension is complicated by proteinuria, maternal organ dysfunction and/or fetal growth restriction [9]. HELLP syndrome causes severe maternal metabolic, vascular, and thrombotic complications. Although maternal mortality in the Netherlands is low, PE/HELLP remains the number one cause [10].

Many women with a history of PE/HELLP remain undiagnosed for their hypertension, since female-specific risk factors are not routinely accounted for in the SCORE or other cardiovascular risk charts. These young women leading busy lives are not likely to seek medical attention to check their blood pressure regularly, and they do not want to be considered as ‘patients’ lifelong either. Therefore, elevated blood pressure may remain undetected and untreated for many years. These considerations can be surpassed using self-monitoring applications.

The aim of our study is to assess whether home blood pressure monitoring (HBPM) in women with a previous history of PE/HELLP is a valuable tool in clinical practice for early detection and timely treatment of hypertension.

### Premature endothelial dysfunction after PE/HELLP

The etiology of PE is still largely unknown. It is hypothesized that PE/HELLP is a result of poor placentation causing a widespread inflammatory response resulting in generalized endothelial dysfunction in the placental and maternal circulation [11, 12]. A balanced immune response is necessary in normal pregnancy for adequate placentation, but the immune response is exaggerated in PE/HELLP. This overreactive inflammatory response is characterized by an increase in pro-inflammatory T lymphocytes and a decrease in regulatory T cells, leading to elevated pro-inflammatory factors, such as cytokines, leukocytes, and adhesion molecules [13, 14]. This results in endothelial dysfunction in the placenta and maternal circulation. It is hypothesized that PE may be the result of a preexistent elevated CVD risk in the mother [15, 16].

Evidence is increasing that maternal endothelial dysfunction persists after such a complicated pregnancy, increasing the risk of hypertension and end-organ damage at young age [17, 18]. Furthermore, there is compelling evidence that an enhanced inflammatory system is a major contributor to atherosclerosis and cardiovascular disease [19–21]. Both PE/HELLP and the development of atherosclerotic disease share several common pathophysiological pathways.

### Hypertension after PE/HELLP syndrome

In the last two decades, a large body of evidence has shown that women after PE/HELLP are at increased risk to develop hypertension before the age of 55 years. In a meta-analysis by Bellamy et al., a relative risk of 3.70 (95% confidence interval (CI) 2.70–5.05) was found for hypertension after PE [22]. We showed previously that 43% of women after early PE had hypertension before the age of 40 years, compared to 17% in a control group of women after a normotensive pregnancy [23]. Behrens et al. demonstrated that women with HDP had a 30% risk of post-pregnancy hypertension, especially in the first 1–2 years [24]. Several international guidelines have now recognized HDP as an important CVD risk factor and recommend screening and management of emerging risk factors in affected women [5, 25]. The excess CVD risk after HDP might be associated with traditional modifiable cardiovascular risk factors, such as elevated blood pressure [26]. However, it remains to be established how, when, and by whom the blood pressure of these young women should be monitored optimally.

### eHealth /home blood pressure monitoring

Up to half of an individual’s CVD risk is attributable to high blood pressure, which makes timely diagnosis of hypertension very important [27]. In the latest 2018 European Society of Cardiology/European Society of Hypertension (ESC/ESH) guideline for the management of arterial hypertension [28] and the 2017 American College of Cardiology/American Hypertension Association (ACC/AHA) guideline on high blood pressure [29], thresholds for diagnosis and treatment of hypertension have shifted towards lower normal blood pressure values. There are various ways to check blood pressure, e.g. in-office BP measurement, home blood pressure monitoring (HBPM), and 24-h ambulatory blood pressure monitoring (ABPM). Nowadays, HBPM is complementary to in-office blood pressure measurements and ABPM in the diagnosis and treatment of hypertension.

HBPM is a simple way to check an individual’s usual blood pressure and has better reproducibility than ABPM [30]. Several advantages of HBPM are that it is easy to use and easy to learn and it is not necessary to visit a general practitioner or hospital, which makes it accessible and relatively inexpensive [31]. HBPM can also distinguish between white coat hypertension and masked hypertension. In a systematic review by Stergiou et al. HBPM was assessed as being as reliable as ABPM in the diagnosis of hypertension [32]. This fits nicely into the current era of modern eHealth applications and personalized medicine with the patient-as-partner in treatment [33]. Another advantage of more intense patient involvement is the higher motivation for lifestyle adherence and better intake of prescribed medication [34]. Since women with a history of PE/HELLP have a life-time elevated CVD risk, self-assessment of blood pressure provides a window of opportunity for timely preventive measures.

Using HBPM, hypertension is diagnosed if the average blood pressure of twice-daily measurements for at least 3, but preferably 7, days is ≥135/85 mmHg [28, 29, 31]. It has not been established yet, however, whether self-measurement of blood pressure may be more effective and feasible for prospective follow-up of women with an elevated CVD risk due to previous PE/HELLP, than incidental blood pressure measurements at a physician’s office. We hypothesize that HBPM will identify women with elevated blood pressure more timely than usual care.

## Methods

### Study design and participants

The feasibility and usability of HBPM to detect hypertension will be evaluated in a multicenter randomized controlled trial (RCT). Participants are women aged 40 to 60 years with a history of PE/HELLP. Seven departments of Obstetrics in the Euregio Rijn-Waal, in The Netherlands and Germany, participate as recruiting centers. All study visits will be executed at the department of Cardiology at the Radboudumc, the principal study center. The databases of the seven departments of Obstetrics were searched for patients with PE and/or HELLP in their medical history. Previous PE/HELLP was defined as diastolic blood pressure (BP) ≥ 90 mmHg and/or systolic BP ≥ 140 mmHg and proteinuria ≥300 mg/24 h, more than 1 year ago. Both patients with early-onset PE (i.e. delivery before 34 weeks of gestation) and late-onset PE (i.e. delivery from 34 weeks of gestation onwards) will be included, as well as patients after eclampsia or HELLP syndrome. Exclusion criteria are previous cardiovascular events (such as myocardial infarction, stroke, documented ischemic heart disease), already diagnosed hypertension and/or use of antihypertensive medication, not having a smartphone, or inability to perform self-measurement of blood pressure. Pregnant women or women aiming to have another pregnancy will be excluded. All eligible patients are invited to participate by letter. In case no response is received after two weeks, we will call the patient to ask for participation. The participating women are randomized to HBPM (intervention group) or ‘usual care’ (control group) using the randomization method in Castor EDC [35]. Patients in the usual care group will not receive planned blood pressure measurements, but they will be asked to register their blood pressure if a measurement is done at a doctor’s visit.

### Data collection

At the baseline visit, the randomization and a physical examination will be conducted consisting of measurements of blood pressure, heart rate, weight, height, and waist and hip circumference. Standardized blood pressure measurements will be performed on both arms, with at least 3 different readings. Waist and hip circumference will be measured with a non-stretchable tape measure over light clothing: waist circumference at the narrowest point of the abdomen and hip circumference at the widest diameter around the buttocks. Finally, a non-fasting blood sample (13 mL) will be drawn. The blood samples will be centrifuged at 1500G for 15 min within 4 h, separated into equal volumes (250 μL), and frozen immediately at minus 80 °C. These samples will be stored for future biomarker analysis.

All participants will also be asked to complete structured online questionnaires at several occasions during follow-up. At baseline, information will be obtained on personal medical history, family history of CVD, obstetric history, cardiovascular symptoms, menopausal symptoms, and lifestyle (e.g. physical activity, dietary patterns, and smoking). In addition, several standardized questionnaires will be administered, such as the 36-item Short Form Health Survey (SF-36), the Copenhagen Psychosocial Questionnaire (COPSOQ-II) on working ability [36], and questionnaires on need for recovery [37] and work-related recovery opportunities [38]. During follow-up, all participants will be asked to complete an online questionnaire on psychosocial well-being (SF-12) every month. The intervention group will also be asked to complete a questionnaire on feasibility and usability of the blood pressure device after 6 months and 1 year of follow-up. At the final visit after 1 year, all baseline measurements and questionnaires will be repeated in both groups.

### Home blood pressure monitoring (HBPM)

HBPM will be performed using the Withings BPM device. This device was validated for home blood pressure measurements according to the ESH International Protocol [39]. The Withings BPM device is a wireless blood pressure monitor, which connects to an application on the woman’s smartphone using Bluetooth. Measurements uploaded to the application will automatically be entered into an online patient health file (Patients Know Best®), which is accessible for the study coordinators to check the blood pressure levels regularly. The participants will be instructed to perform the blood pressure measurements according to the ESH guideline [31] for blood pressure home monitoring. These guidelines advise BP measurements in the morning and in the evening, at least two measurements per occasion, preferably over 7 consecutive days. Currently, HBPM is only used for self-management in poorly controlled hypertension [40–44], but not yet to detect hypertension in high-risk patients. As a result, the guidelines do not describe the frequency of the measurement periods. For this study, the participants will be asked to install a 7-days measurement period every month during 1 year.

### Outcomes

The primary outcome is the feasibility and usability of HBPM during 1 year of follow-up. The participants in the intervention group will receive monthly feedback on their blood pressure measurements. In case of elevated blood pressure, participants will be advised to contact their general practitioner for treatment advice. Feasibility and usability as well as protocol adherence and persistence to HBPM will be determined using the questionnaires completed after 6 months and 1 year of follow-up and the actual BP measurements. Protocol adherence is the proportion of participants in the intervention group who self-monitor their blood pressure twice daily for at least 4 consecutive days every month during 1 year of follow-up. Persistence to HBPM is defined as the proportion of participants in the intervention group who measure their blood pressure until the final follow-up visit. The secondary outcomes include blood pressure levels and the prevalence of hypertension in the intervention group compared to the control group at 1 year of follow-up. In addition, quality of life, work capability, and cardiac and/or hypertensive symptoms will be compared between the groups.

### Sample size calculation

Formally, a sample size calculation is not required for a feasibility study. The prevalence of PE/HELLP in the Dutch/German population is 3–5% of all pregnancies per year. Among 5.5 million inhabitants in the study region as a whole, we expect approximately 55,000 pregnancies each year, among which an estimated 2200 are complicated by PE/HELLP. If the participating obstetric centers (out of 28 hospitals in the region) cover approximately 20% of these pregnancies, more than 4000 women with PE/HELLP should have been seen in the past 10 years. These women can be considered our study base from which the study participants can be recruited.

Within the time-frame and the financial constraints of the study, it would be realistic to recruit approximately 200 eligible women, with inclusion of 100 women in the intervention group and 100 women in the usual care group. For the secondary endpoint BP, we would be able to find differences of approximately 5 or more mmHg in SBP (assuming a SD of 12 mmHg) and 4 or more mmHg in DBP (with a SD of 9 mmHg) with 80% power and a 0.05 level of significance (two-sided), assuming less than 10% loss-to-follow-up. In comparison to other self-monitoring of blood pressure studies, a difference of 5 mmHg in systolic blood pressure is plausible [45].

### Statistical analysis

Data analysis will be performed using SPSS (IBM). The characteristics of the participants will be described using means with 95% confidence intervals for normally distributed continuous variables or medians plus ranges otherwise, and proportions for categorical variables. Both qualitative and quantitative analyses will be performed on the primary and secondary outcomes with a range of statistical techniques to compare the baseline data with the 6 months and 1 year follow-up data taking into account study group. To describe protocol adherence and persistence to HBPM, survival analyses will be performed.

### Ethical considerations

This study was approved by the Regional Committee on Research involving Human Subjects Arnhem-Nijmegen (number 2016–3006). This trial was registered in the Clinical Trials Register, NCT03228082, https://clinicaltrials.gov/ct2/show/NCT03228082, date of registration: June 15, 2017.

## Discussion

We aim to demonstrate that HBPM is an acceptable and easy way to check blood pressure regularly. We assume that by using HBPM, the blood pressure in de intervention group will be regulated better after 1 year of follow-up compared to the control group. By including the patient actively in HBPM, we expect to be able to diagnose hypertension earlier and to establish more timely treatment. If HBPM shows to be a feasible method to diagnose hypertension in the high-risk women included in this study, this method may be useful for follow-up starting directly after pregnancies complicated by hypertensive disorders. The incidence of hypertension among women with these pregnancy complications is expected to be even higher than the findings of this study will show, since we exclude women with already diagnosed hypertension. A limitation is that we cannot confirm the diagnosis hypertension using ABPM for all women in the study. However, recent studies showed a diagnostic agreement ranging from 80 to 90% between the two methods [46]. Therefore, we expect that HBPM is a valuable tool for blood pressure monitoring for long-term follow-up.

## Data Availability

Not applicable.

## Abbreviations

ABPM: Ambulatory blood pressure monitoring
ACC: American College of Cardiology
AHA: American Hypertension Association
BP: Blood pressure
CI: Confidence interval
COPSOQ-II: Copenhagen Psychosocial Questionnaire
CVD: Cardiovascular disease
ESC: European Society of Cardiology
ESH: European Society of Hypertension
HBPM: Home blood pressure monitoring
HDP: Hypertensive disorders of pregnancy
HELLP: Hemolysis, elevated liver enzymes, low platelets
PE: Preeclampsia
PIH: Pregnancy-induced hypertension
RCT: Randomized-controlled trial
RR: Relative risk
SF-12: 12-item Short Form Health Survey
SF-36: 36-item Short Form Health Survey
